# Testing the validity of the proposed ICD-11 PTSD and complex PTSD criteria using a sample from Northern Uganda

**DOI:** 10.3402/ejpt.v7.32678

**Published:** 2016-09-08

**Authors:** Siobhan Murphy, Ask Elklit, Sarah Dokkedahl, Mark Shevlin

**Affiliations:** 1 Department of Psychology, National Centre of Psychotraumatology, University of Southern Denmark, Odense, Denmark; 2 Psychology Research Institute, Ulster University, Coleraine, UK

**Keywords:** ICD-11, PTSD, complex PTSD, latent class analysis, Northern Uganda

## Abstract

**Background:**

The International Classification of Diseases (ICD-11) is currently under development with proposed changes recommended for the posttraumatic stress disorder (PTSD) diagnosis and the inclusion of a separate complex PTSD (CPTSD) disorder. Empirical studies support the distinction between PTSD and CPTSD; however, less research has focused on non-western populations.

**Objective:**

The aim of this study was to investigate whether distinct PTSD and CPTSD symptom classes emerged and to identify potential risk factors and the severity of impairment associated with resultant classes.

**Methods:**

A latent class analysis (LCA) and related analyses were conducted on 314 young adults from Northern Uganda. Fifty-one percent were female and participants were aged between 18 and 25 years. Forty percent of the participants were former child soldiers (*n*=124) while the remaining participants were civilians (*n*=190).

**Results:**

The LCA revealed three classes: a CPTSD class (40.2%), a PTSD class (43.8%), and a low symptom class (16%). Child soldier status was a significant predictor of both CPTSD and PTSD classes (OR=5.96 and 2.82, respectively). Classes differed significantly on measures of anxiety/depression, conduct problems, somatic complaints, and war experiences.

**Conclusions:**

To conclude, this study provides preliminary support for the proposed distinction between PTSD and CPTSD in a young adult sample from Northern Uganda. However, future studies are needed using larger samples to test alternative models before firm conclusions can be made.

**Highlights of the article:**

- Examine the validity of CPTSD in a non-western sample
- Separate PTSD and CPTSD classes emerged
- Former child soldiers were more strongly associated with the CPTSD class
- CPTSD class reported significantly higher levels of anxiety, depression, somatic complaints and conduct problems

The International Classification of Diseases (ICD) has evolved through 10 editions with the ICD-11 due for publication in 2018 by the World Health Organization. In light of its release, a series of articles have been published outlining some proposed revisions to disorders specifically associated with stress (Maercker et al., 2013a, b). Recommendations have been made for the diagnostic criteria for posttraumatic stress disorder (PTSD) to have a narrower definition characterised by some degree of fear or horror with clearly distinguishable symptoms to other psychiatric conditions (Cloitre, Garvert, Brewin, Bryant, & Maercker, 2013). It is also intended to direct clinicians to the core elements of the disorder and use functional impairment rather than a specific traumatic experience to determine diagnostic threshold (Maercker et al., 2013a). The proposed reformulation of PTSD contrasts significantly to the 20 symptoms in the *Diagnostic and Statistical Manual of Mental Disorders*, fifth edition, PTSD criteria (DSM-5; American Psychiatric Association, 2013). The *DSM*-5 criteria of PTSD include four symptom clusters: intrusions, avoidance, negative alternations in cognitions and mood, and alternations in arousal and reactivity. The suggested ICD-11 criteria include two symptoms of re-experiencing of the traumatic event(s) in the present accompanied by emotions of fear or horror, two symptoms of avoidance of traumatic reminders, and two symptoms representing a sense of current threat (excessive hyper vigilance or an enhanced startle reaction).

Another proposed revision to the ICD-11 is the addition of complex PTSD (CPTSD; Cloitre et al., 2013; Maercker et al., 2013a, b) and the removal of “Enduring personality change after catastrophic experience.” CPTSD was initially introduced by Herman (1992) and manifests following prolonged and repeated traumatic events from which separation is not possible (e.g., war captivity, genocide, and childhood sexual abuse). CPTSD consists of the three core features of PTSD in addition to difficulties in affect dysregulation, self-concept, and relational functioning, collectively described as disturbances in self-organisation (DSO). A diagnosis of CPTSD requires that in addition to the PTSD symptoms, an individual must display at least one symptom from each of DSO domains (Maercker et al., 2013b). Affect dysregulation consists of a range of symptoms resulting from difficulties in emotion regulation which may manifest in heightened emotional reactivity (hyper-activation) or in a lack of emotions or dissociative symptoms (deactivation). Self-concept difficulties refer to persistent negative beliefs about oneself, feelings of worthlessness, shame, and guilt. Disturbances in relational functioning are characterised by difficulties in feeling emotionally close or engaging with others. A distinguishable feature of the two trauma-related disorders is that PTSD symptoms are related to the trauma-specific stimuli, whereas DSO symptoms are ubiquitous and occur across various contexts and relationships regardless of proximity to traumatic reminders (Cloitre et al., 2013).

Before implementing a new diagnosis, the validity and clinical utility must be empirically tested across different settings. Cloitre et al. (2013) investigated the distinctiveness of the proposed ICD-11 criteria of PTSD and CPTSD using a sample of 302 individuals exposed to both chronic (e.g., childhood abuse) and single-incident (e.g., 9/11 exposure) traumatic events. Latent profile analysis revealed a three-class model. A “CPTSD” class (36.1%) was characterised by elevated PTSD and DSO symptoms. A “PTSD” class (31.8%) was characterised by elevated levels of PTSD but low endorsement of DSO symptoms. The third class was characterised by low level of endorsement across all items and was labelled a “low symptom” class (32.1%). Consistent with the theoretical framework of CPTSD, the authors found prolonged trauma (e.g., childhood abuse) was predictive of CPTSD compared to PTSD whilst 9/11 exposure was predictive of PTSD as opposed to CPTSD. The authors further noted that the PTSD and CPTSD classes did not differ in terms of severity of PTSD symptoms and that the CPTSD class was associated with greater functional impairment than the PTSD class.

Elklit, Hyland, and Shevlin (2014) extended these findings using three independent trauma samples (*N*=1,251) consisting of bereaved parents, sexual assault survivors, and physical assault survivors. This study utilised latent class analysis (LCA) which identifies classes based on categorical data, as opposed to the continuous multivariate data that are used in latent profile analysis. Three distinct classes emerged that were a “CPTSD” class, a “PTSD” class and a “low symptom” class for each of the trauma samples. The findings indicated that a higher proportion of the sexual trauma sample was in the CPTSD class (20.7%) compared to physical assault and bereaved parents’ samples (13% and 10.4% respectively). Higher levels of functional and psychological impairment were evident in the CPTSD classes supporting the findings of Cloitre and colleagues study.

Further evidence was provided by Knefel, Garvert, Cloitre, and Lueger-Schuster (2015) who conducted a latent profile analysis on a predominantly male sample exposed to childhood institutional abuse (*N*=229). They reported a four-class model comprised of a “CPTSD” class (20.1%), a “PTSD only” class (17.5%), a “DSO” class (19.2%), and a class with low symptoms (43.2%). Importantly, the findings from the above-mentioned studies support the distinction of PTSD and CPTSD that has been proposed by the upcoming ICD-11 and have tested the validity of this proposition in a range of trauma samples.

However, Wolf et al. (2015) reported contrary findings using a community sample (*N*=2,695) and a sample of trauma-exposed military veterans (*N*=323). Applying latent trait, latent class, and hybrid models, they found a two-dimensional four-class model best fitted the data. The results indicated that classes were differentiated on the basis of severity of symptoms with individuals with high levels of PTSD also reporting high CPTSD and those with low levels of PTSD reporting low levels of CPTSD. Further trauma history was not predictive of class membership. Notably, the authors acknowledged that when conducting only a latent profile analysis, the findings revealed similar models to the aforementioned studies; however, a different pattern emerged when factor mixture modelling was applied. They concluded that additional research is needed to determine how to conceptualise CPTSD symptoms in relation to PTSD. Further, in a sample of West Papuan refugees, Tay, Rees, Chen, Kareth, and Silove (2015) found support for the proposed ICD-11 PTSD criteria but failed to find support for DSM-IV, DSM-5 PTSD, and ICD-11 criteria for CPTSD.

Collectively, whilst some empirical evidence supports the proposed ICD-11 model of separate PTSD and CPTSD disorders in western populations (Cloitre et al., 2013; Elklit et al., 2014; Knefel et al., 2015), CPTSD has yet to be validated in non-western samples. The current study intends to contribute to empirical research by using data from a young adult population from Northern Uganda. The civil war in Northern Uganda between the Ugandan government forces and the Lord's Resistance Army (LRA) lasted 22 years and ended in 2006. The war resulted in serious human rights violations on the population as a whole, cost the lives of approximately 200,000 people and increased poverty across the country (Klasen, Reissmann, Voss, & Okello, 2015). Over 2 million people were internally displaced and many of civilians predominantly children were abducted and used as child soldiers in the LRA (Mugisha, Muyinda, Wandiembe, & Kinyanda, 2015). The prevalence of PTSD in Northern Uganda varies substantially ranging from 11 to 54% (Mugisha et al., 2015; Roberts, Ocaka, Browne, Oyok, & Sondorp, 2008). Variation in prevalence estimates may be the result of the different measures to assess PTSD and the time the data were collected (e.g., when active conflict was ongoing) which could result in the overestimation of PTSD and/or possible misdiagnosis of other traumatic reactions.

There have been many identified risk factors for PTSD in war affected populations such as gender (Ai, Peterson, & Ubelhor, 2002; Betancourt et al., 2013; Johnson & Thompson, 2008; Roberts et al., 2008) and cumulative war trauma experiences (Ai et al., 2002; Rosner, Powell, & Butollo, 2003). Another risk factor that has attracted research attention in terms PTSD is child soldier status (Betancourt et al., 2013). It has been estimated that despite international bans, more than 250,000 children and adolescents are exploited as child soldiers worldwide, with a higher prevalence reported in Africa (Coalition to Stop the Use of Child Soldiers, 2008). Studies investigating child soldiers have demonstrated the range of traumatic experiences endured such as physical torture, extreme deprivation, sexual abuse, and being forced to commit atrocities against each other and their own communities including killing family members, raids on villages and looting (Amone-P'Olak et al., 2013; Mugisha et al., 2015).

The first aim of the study was to examine the distinctiveness of PTSD and CPTSD in a non-western population exposed to community rather than individual trauma. It is hypothesised that if there is a distinction between PTSD and CPTSD, an LCA would produce a two- or three-class solution, differing qualitatively: a two-class solution would have a PTSD and CPTSD class, and a three-class solution would have an additional “baseline” class with relatively low probabilities of endorsing any symptom as evidenced in previous studies (Cloitre et al., 2013; Elklit et al., 2014). Alternatively, if there is no distinction between PTSD and CPTSD, then it is predicted that the LCA would produce a number of classes that differ only quantitatively thereby representing arbitrary cut-points on a PTSD/CPTSD continuum. The second aim was to examine the association between demographic and trauma-related variables (gender, and abduction by the LRA) and the resultant classes. Previous studies have identified female gender as predictive of PTSD (Johnson & Thompson, 2008; Roberts et al., 2008); therefore, it was hypothesised that female gender would confer a higher risk of disorder compared to a baseline class. It was also hypothesised that child soldier status would confer a higher risk for PTSD and CPTSD than baseline classes if they emerged. The third aim was to examine the resultant latent classes as predictors of a range of mental health outcomes and prosocial behaviour. It is hypothesised that those classes who displayed higher levels of CPTSD would exhibit a higher probability of comorbid symptomatology consistent with previous studies (Cloitre et al., 2013; Elklit et al., 2014).

## Methods

### Sample

This study was conducted in Gulu, in the subcounty of Awach, the biggest district of Northern Uganda. The sample (*N*=314) included both males (49%) and females (51%) aged between 18 and 25 years. Forty percent (39.5%) of the participants were abducted by the LRA (*n*=124) while the remaining participants were civilians (*n*=190).

The lack of a national register in Uganda made random sampling difficult. The local leaders of the four parishes in Awach were informed of the study and selected the participants based on the principles of random sampling and in accordance to the size of the parish. Participants were further selected equally from each village within the parish with equal participation between gender, age, former child soldiers, and civilians. This allowed for a broad distribution of participants within the Awach community. Exclusion criteria were presence of psychotic symptoms or for individuals who were unable to complete the interview due to mental disability.

Participants gave written consent for their participation and those who could not write were asked to sign with their thumbprint in ink. Before giving consent the participants were informed about the content of the study, their rights to decline and withdraw at any time, and the confidentiality of their participation in the study. None of the participants who were selected declined to participate. As per agreement with Victim's Voice (VIVO), participants who fulfilled the criteria for PTSD, using the ICD-11 measure, and who wished to receive help were referred for counselling at VIVO. Participants were informed that the doctor at the local health centre had agreed that all participants could seek help and support at the health centre if necessary. The Institutional Review and Ethics Committee at the Lacor Hospital in Gulu approved the project along with the translations. All measures and instruments were translated and back-translated from English into Luo the local language of the Awach community.

The questionnaires were read out loud for the participants to avoid any possible reading disabilities in the rural areas of Northern Uganda. Local field assistants asked the questions in the local language, Luo, which is the language spoken by the Acholi tribe. The interviews took place at the homes of the participants.

### Measures

#### UNICEF War Trauma Screening Scale

Trauma exposure was measured using the UNICEF War Trauma Screening Scale, which was originally developed for Bosnia and Herzegovina but has been adapted for use in African war-affected youth (Amone-P'Olak et al., 2013; UNICEF, 2010). The instrument consists of items relating to personal injury (6 items), witnessing violence (11 items), injuries and threats to self (5 items), deaths (7 items), physical threats to loved ones (4 items), material losses (4 items), harm to loved ones (4 items), separation (2 items), displacement (5 items), participating in armed groups (4 items), and sexual abuse (3 items). In the current study, the reliability estimate for the total scale was high with *α*=0.93.

#### ICD-11 Trauma Questionnaire (ICD-TQ Version 1.4)

The ICD-TQ is a 23-item self-report measure for ICD-11 PTSD and CPTSD diagnoses that is currently under development (Cloitre, Roberts, Bisson, & Brewin, 2015). The measure corresponds to the three clusters of PTSD including Re-experiencing (RE) (items P1–P2), Avoidance (AV) (items P3–P4), and Sense of Threat (Th) that is manifested by increased arousal and hyper-vigilance (items P5–P6). CPTSD is measured through the inclusion of 16 symptoms that capture DSO symptoms. These items include four clusters with two relating to affect regulation (AR) characterised by hyper-activation (C1–C5) or deactivation (C6–C9), negative self-concept (NSC; C10–C13), and disturbed relationships (DR; C14–C16).

The response format corresponds to the degree the symptoms bothered the individual in the past month and are scored on a Likert scale ranging from 0 (not at all) to 4 (extremely). The scale can be used to generate a self-report ICD-11 PTSD or CPTSD diagnosis. A diagnosis of PTSD requires a score of ≥2 for at least one symptom in each of its three clusters. A diagnosis of CPTSD requires PTSD and the following scores for each of the three DSO clusters. AR for consistency requires a score ≥10 on items 1–5 (hyper-activation) or a score of ≥8 on items 6–9 (deactivation); for the NSC items a score ≥8 and for DR a score ≥6 are required. Binary variables were created based on meeting the criteria for each cluster.

In the current study, the reliability estimates were adequate with Cronbach's alpha for the total scale (*α*=0.91), hyper-activation (*α*=0.73), deactivation (*α*=0.75), negative self-concept (*α=*0.83), and relational disturbance (*α=*0.79).

#### African Youth Psychosocial Assessment

Mental health was assessed by the African Youth Psychosocial Assessment Instrument (APAI), which is a field-based instrument developed for use in Northern Uganda (Betancourt et al., 2009). This measure comprises four subscales of depression/anxiety (18 items), somatic complaints (3 items), conduct problems (10 items), and prosocial behaviour (5 items). The instrument measures on a 4 point Likert scale ranging from 0 (not at all) to 3 (always). The reliability in the current sample was adequate with Cronbach's alpha values anxiety/depression (*α*=0.91), conduct problems (*α*=0.81), somatic complaints (*α*=0.66), and prosocial behaviour (*α*=0.64).

### Analysis

LCA (e.g., McCutcheon, 1987) is a statistical method used to identify homogeneous groups, or classes, from multivariate categorical data. LCA was conducted to determine the nature and number of classes based on PTSD and DSO subscales. The fit of six models (one- through six-class model) was assessed. The models were estimated using robust maximum likelihood (Yuan & Bentler, 2000). To avoid solutions based on local maxima, 100 random sets of starting values were used initially and 50 final stage optimisations. The relative fit of the models were compared using three information theory based fit statistics: the Akaike Information Criterion (AIC; Akaike, 1987), the Bayesian Information Criterion (BIC; Schwarz, 1978), and sample size adjusted Bayesian Information Criterion (ssaBIC; Sclove, 1987). The model that produces the lowest values can be judged the best model. Evidence from simulation studies has indicated that the BIC was the best information criterion for identifying the correct number of classes (Nylund, Asparouhov, & Muthen, 2007). In addition, the Lo–Mendell–Rubin adjusted likelihood ratio test (LMR-A; Lo, Mendell, & Rubin, 2001) and the bootstrapped likelihood ratio test (BSLRT; McLachlan & Peel, 2000) were used to compare models with increasing numbers of latent classes. When a non-significant value (*p*>0.05) occurs this suggests that the model with one less class should be accepted. All analyses were conducted using Mplus 7.1 (Muthen & Muthen, 1998–2012). A logistic regression was conducted to assess whether gender and LRA status predicted class membership. Finally, the resultant classes were used as independent variables in one way analysis of variances (ANOVAs) with the APAI subscales and Total War Experiences as the dependent variables. Pairwise comparisons using Tukey were conducted to examine significant differences between classes.

## Results

A total of 33.9% (*n*=106) of the sample met the ICD-11 criteria for PTSD and 20.8% (*n*=65) met the criteria for CPTSD using the ICD-11 TQ. Chi square analysis indicated no significant difference between gender and PTSD (*χ*2 (1)=2.05, *p*>0.05) or gender and CPTSD (χ2 (1)=1.85, *p*>0.05). Table 1 shows the frequencies of PTSD and DSO items using the binary variable format. For the total sample, items relating to PTSD re-experiencing symptoms and emotion regulation (specifically symptoms relating to hyper-activation) were the most commonly endorsed experiences, for example, reporting episodes of uncontrollable anger and taking a long time to calm down. Symptoms of PTSD internal avoidance and avoidance of relationships and feeling numb were the least commonly endorsed items.

**Table 1:** PTSD and CPTSD endorsement rates

ICD-11	Item	*n*	%
P1 Re-Exp	Upsetting dreams that replay part of the event or are clearly related to the event?	202	64.5
P2 Re-Exp	Powerful images or memories that sometimes come into your mind in which you feel the event is happening again in the here and now?	145	46.3
P3 Avoid	Avoiding internal reminders of the stressful event experience (for example, thoughts, feelings, or physical sensations)?	123	39.4
P4 Avoid	Avoiding external reminders of the stressful event experience (for example, people, places, conversations, objects, activities, or situations)?	142	45.8
P5 Arousal	Being “super-alert,” watchful, or on guard?	125	40.1
P6 Arousal	Feeling jumpy or easily startled?	133	42.5
C1 Hyper	I react intensely to things that don't seem to affect other people so much?	155	51.3
C2 Hyper	When I am upset, it takes me a long time to calm down.	184	59.9
C3 Hyper	My feelings tend to be easily hurt.	191	62.2
C4 Hyper	I experience episodes of uncontrollable anger.	185	61.3
C5 Hyper	I do things that people have told me are dangerous or reckless (for example, driving very fast).	192	63.4
C6 Deact	I feel numb or emotionally shut down.	77	24.8
C7 Deact	I am the kind of person who has difficulty experiencing feelings of pleasure or joy.	154	50.7
C8 Deact	When I am under stress or confronted with reminders of my trauma, I often feel that the world is distant or that the world seems different (for example, time slows down, things look different).	177	59.2
C9 Deact	When I am under stress or confronted with reminders of my trauma, I often feel outside my body or feel that there is something strange about my body.	167	54.2
C10 SC	I feel like a failure.	160	42.4
C11 SC	I feel worthless.	139	45.9
C12 SC	I often feel ashamed of myself whether it makes sense or not.	141	46.1
C13 SC	I feel guilty about things I have done or failed to do.	187	60.3
C14 DR	I feel distant or cut off from people.	152	49.0
C15 DR	I find it hard to stay emotionally close to people.	126	40.4
C16 DR	I avoid relationships because they end up being too difficult or painful.	108	34.6

The fit statistics for the LCA analyses based on the PTSD and CPTSD subscales are reported in Table 2. The model fit indices indicate a three-class model best fitted the data. Support for a three-class model was evidenced through a non-significant LMR-A and BSLRT in a four-class solution which indicates the model with one less class should be selected. Additionally, the information criterion indices AIC, BIC, and ssaBIC all are lowest for a three-class model. Therefore, a three-class model was considered the best solution and indicated good class discrimination with high probabilities for most likely latent class membership (class 1=0.96; class 2=0.91; class 3=0.90).

**Table 2:** Fit indices from latent class models

Classes	Log	AIC	BIC	ssaBIC	LMR-A (p)	BSLRT	Entropy
1	−1433.02	2880.03	2906.26	2884.05	–	–	–
2	−1195.30	2420.60	2476.79	2429.21	465.31 0.00	475.44 0.00	0.87
**3**	−**1169.81**	**2385.62**	**2471.79**	**2398.84**	**49.89** **0.00**	**50.97** **0.00**	**0.82**
4	−1163.31	2388.61	2504.74	2406.42	12.74 0.55	13.01 0.67	0.83
5	−1157.48	2392.95	2539.05	2415.36	11.41 0.47	11.65 1.00	0.81
6	−1152.72	2399.45	257.52	2426.45	9.30 0.21	9.50 1.00	0.84

*Note*: Best fitting model highlighted in bold, Log=Log likelihood, AIC=Akaike information criterion, BIC=Bayesian information criterion, ssaBIC= sample-size adjusted BIC, LMR-A LRT= Lo–Mendell–Rubin adjusted likelihood ratio test, BSLRT=Bootstrapped likelihood ratio test.

**Fig 1:**
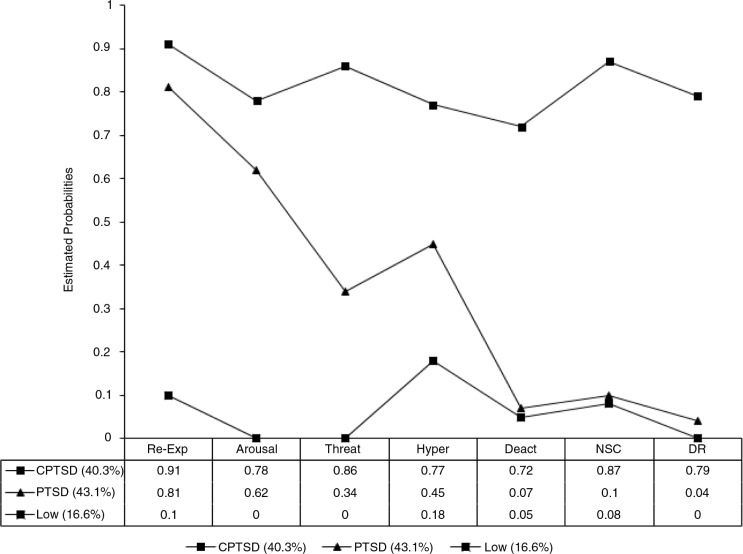
Profile plot of estimates from latent class analysis of CPTSD items.

Figure 1 shows the profile plot for the three-class solution. Class 1 (*n*=126; 40.3%) was characterised by a high probability of having experienced both PTSD and DSO symptoms. This class was labelled as the CPTSD class. Class 2 (*n*=135; 43.1%) was the largest class and was characterised by high levels of PTSD symptoms, moderate levels of hyper-activation items but low levels of deactivation, negative self-concept, and disturbed relationships. This class was labelled the PTSD class. Class 3 (*n*=52; 16.6%) was the smallest class and was characterised by low endorsement of PTSD and CPTSD items and therefore was labelled the low symptom class. Notably, this class did report elevated hyper activation scores but at attenuated levels in comparison to the CPTSD and PTSD classes.

Two independent variables (being female and abducted by the LRA) were entered into a multinomial logistic regression with the latent classes utilised as the dependent variable. Results indicated that being female was not a significant predictor of class membership. Child soldier status was a significant predictor of class membership for the CPTSD class (OR=5.43; 95% CI, 2.44–12.09) and PTSD class (OR=2.54; 95% CI, 1.14–5.67) relative to the low symptom class.

A series of one-way ANOVAs were conducted to examine differences in class membership on their mean scores using the APAI subscales and total number of traumatic events. Table 3 presents the findings of this part of the analysis. The results indicated that the CPTSD class reported the highest levels of anxiety and depression, conduct problems, and somatic complaints and differed significantly from the PTSD class and the low symptom class across all variables. The PTSD class also differed significantly from the CPTSD and low symptom class with the exception of conduct problems. There were no significant differences between the classes and levels of prosocial behaviour. In terms of war experiences, the CPTSD class reported significantly higher levels of exposure than the other classes with an overall mean of 38 different types of war traumatisation experiences, followed by the PTSD class and low symptom class.

**Table 3:** Results of univariate ANOVAs using class membership as the independent variable and APAI symptom clusters and total war experiences as dependent variables

	CPTSD (40.3%)	PTSD (43.1%)	Low (16.6%)	
		
	M (SD)	M (SD)	M (SD)	F
Anxiety–depression	30.28^a^ (8.48)	18.78^b^ (7.93)	13.04^c^ (7.89)	106.52***
Conduct	6.18^a^ (5.52)	3.90^b^ (3.57)	3.88^b,c^ (5.13)	8.86***
Somatic	5.61^a^ (2.11)	3.97^b^ (2.17)	2.82^c^ (3.97)	39.09***
Prosocial	9.55^a^ (2.78)	9.76^a^ (2.19)	9.71^a^ (1.85)	0.26
Total war experiences	38.41^a^ (7.63)	33.77^b^ (8.47)	27.45^c^ (11.08)	30.39***

*** *p<*0.001. Means with different superscript letters are significantly different at *p*<0.05.

## Discussion

The primary objective of the current study was to investigate the proposed ICD-11 distinction between PTSD and CPTSD using a non-western sample of young adults exposed to war trauma in Northern Uganda. The LCA identified a three-class model characterised by a CPTSD class, a PTSD class, and a low symptom class. However, the findings do not clearly point to the distinction between the two disorders as Fig. 1 illustrates quantitative differences in the severity of PTSD and CPTSD symptoms. For example, the CPTSD class displays high probabilities of endorsing all PTSD and DSO items. The PTSD class conversely, do endorse high levels of re-experiencing and arousal symptoms but moderate levels of sense of threat and hyper activation. The low endorsement of deactivation items and negative self-concept and disturbed relationships in the PTSD class demonstrated a qualitatively distinct profile which could be argued to favour distinguishing between the two separate disorders but further replication is necessary before firm conclusions can be made. Importantly, it should be noted that the current findings differ from previous studies investigating the distinction between PTSD and CPTSD (e.g., Cloitre et al., 2013) as the current symptom clusters incorporated a broader array of DSO items (e.g., difficulty in experiencing positive affect and dissociative symptoms), whereas previous studies were based on item level analyses using only two items for the DSO clusters.

The LCA indicated that the majority of the sample experienced either PTSD or CPTSD with a small minority (16.6%) reporting few or no symptoms. A possible explanation for the presence of such a small low symptom class may be the specific sample used in the study. The severity and range of traumatic experiences which the participants had endured is also a unique characteristic of the sample (in comparison to previous studies in the area) with the high levels of war trauma exposure and the high proportion of former child soldiers. Whatever the explanation, the data have important implications in terms of treatment management in post-conflict societies and the lasting effects of war and violence on the lives of young people. These findings suggest that even a decade since the war in Northern Uganda ended, high levels of traumatic symptomatology are still prevalent. Therefore, investment into treatment interventions and mental health facilities should remain a high priority to support recovery in the communities exposed to mass conflict.

The LCA indicated that 40.3% reported CPTSD which is higher than previous studies with estimates of CPTSD reported as 36% (Cloitre et al., 2013), 10–20.7% (Elklit et al., 2014), and 20% (Knefel et al., 2015). This finding is not surprising however and likely the result of both the civil conflict and post-war factors such as displacement, death and disappearance of family members, land disputes, high levels of poverty, inadequate housing, and healthcare, all of which contribute to ongoing psychological distress in post-war environments (Amone-P'Olak et al., 2014; Miller & Rasmussen, 2010). Further, the participants were children when active conflict was ongoing, and therefore they may be more at risk of CPTSD due to the developmental period in which the trauma occurred.

Importantly, high levels of emotional reactivity characterised by the hyper activation cluster such as “experiencing episodes of uncontrollable anger” and “taking a long time to calm down when feeling upset” were evident in all the classes with markedly elevated levels in the CPTSD class and moderate levels in the PTSD and low symptom class. Numerous studies have noted the strong correlation between PTSD and anger in traumatised populations and that the correlation was significantly higher with increasing time since the event and in military war experiences (Orth & Wieland, 2006). Additionally, other studies highlight that anger is a normative response to injustice in societies that have endured serious human rights violations such as Cambodia (Hinton, Hsia, Um, & Otto, 2003) and Timor-Leste (Rees et al., 2013). This finding has important implications for treatment interventions in post-conflict societies as anger control may be a maintaining factor for persistent PTSD and CPTSD. Support for this assertion was evident in a veteran sample undergoing cognitive therapy whereby moderate and high levels of pre-treatment anger affected the individual's ability to make cognitive reappraisals required to reduce their PTSD symptoms (Owens, Chard, & Cox, 2008). Therefore, targeting feelings of anger in traumatised populations exposed to mass conflict and severe human rights violations should be considered a treatment priority.

The second aim of the study was to assess whether identified risk factors in the literature predicted class membership. Female gender was not found to predict class membership relative to the low symptom class. This finding contradicts other research in similar populations that have found being female is predictive of higher levels of PTSD (Johnson & Thompson, 2008; Roberts et al., 2008). Conversely, other studies have identified a “ceiling effect,” whereby high levels of PTSD following particularly noxious traumas (e.g., sexual assault, combat) in males as well as females will surpass any specific female vulnerability to traumatic stress (Christiansen & Elklit, 2012; Gavranidou & Rosner, 2003).

Child soldier status was found to be highly predictive of class membership with individuals in the CPTSD class being over five times more likely to have been abducted by the LRA relative to the low symptom class. This finding was not unexpected as child soldiers represent a population likely to experience CPTSD due to exposure to a wide range of traumatic experiences such as torture, killings, and sexual abuse at an early age. Child soldiers are also often abducted into armed groups such as the LRA against their wishes and attempts to escape are accompanied by severe physical attacks or death. Former child soldiers have the additional burden of being both a recipient and perpetrator of violence, with studies showing greater difficulties in psychological recovery and reintegration into the community (Bayer, Klasen, & Adam, 2007) which ultimately may impede trauma recovery. Further, Bayer et al. (2007) found that child soldiers with clinically relevant symptoms of PTSD had significantly less openness to reconciliation and significantly more feelings of revenge than those with fewer PTSD symptoms. Although studies have demonstrated the effectiveness of interventions for former child soldiers (e.g., Ertl, Pfeiffer, Schauer, Elbert, & Neuner, 2011) these findings indicate that a decade later this particular group continues to experience high levels of posttraumatic symptomatology.

Finally, the third hypothesis predicting the CPTSD class would report higher levels of comorbid symptomatology than the other classes was supported. Individuals in the CPTSD class reported significantly higher levels of anxiety, depression, somatic complaints, and conduct problems than the other three classes, complementing previous studies that found CPTSD to be associated with higher levels of psychopathology (e.g., Cloitre et al., 2013; Elklit et al., 2014). Additionally, the PTSD class had significantly higher levels of comorbid symptoms than the low symptom class. In terms of total war experiences, both PTSD and CPTSD classes had significantly higher means than the low symptom class. The CPTSD class reported the highest number of war experiences supporting evidence of dose-response relations between cumulative trauma and PTSD in war populations (Ai et al., 2002; Catani, Jacob, Schauer, Kohila, & Neuner, 2008).

The results of this study should be considered in light of some limitations. The ICD-11 TQ has yet to undergo psychometric testing and did not include indicators of functional impairment or duration of symptoms. Further, some studies have found discordant results between self-report measures of PTSD and clinician-administered PTSD scales (Cody, Jones, Woodward, Simmons, & Beck, 2015). Additionally, the threshold used for endorsement of PTSD symptoms is relatively low (rated as present with a score of ≥2) which may account for the high levels of PTSD in the current study. Caution is therefore warranted in interpreting these findings as it is difficult to ascertain whether the findings are specific to the performance of these items in the current sample or the ICD-11 TQ criteria. However, as a validated and standardised measure of CPTSD has yet to be established this limitation was unavoidable. Future research in developing instruments to diagnose and measure symptoms of CPTSD is therefore critical to establish the construct validity of CPTSD (Resick et al., 2012). Another limitation to this study was the lack of random sampling and the selection of participants due to the lack of a national register in Uganda. Also as the current findings are based on a sample of young adults from Northern Uganda it is unknown how they will generalise to other populations. Finally, the current debate within the field on the symptom overlap between CPTSD and borderline personality disorder was not addressed.

In conclusion, the results of the current study do not provide firm support for the distinction between PTSD and CPTSD as separate disorders as proposed in the upcoming ICD-11. However, the emergence of separate CPTSD and PTSD classes differing in terms of DSO symptoms and subsequent comorbid symptomatology provides preliminary support. Replication using a larger sample is warranted to formally test alternative dimensional or hybrid models in order to provide stronger support for the validity of the CPTSD construct. Nevertheless, the study has several strengths. First, there is a paucity of studies using a non-Western sample exposed to mass conflict to test the validity of the proposed ICD-11 PTSD and CPTSD constructs. Second, the findings indicate that despite a decade since the cessation of hostilities in Northern Uganda high levels of PTSD and DSO symptomatology remain. Third, the presence of elevated anger-related symptoms in the majority of the sample suggests that interventions targeting anger-related problems in post-conflict societies are an important component of PTSD recovery.

## Funding statement

This study was funded by the National Center for Psychotraumatology, University of Southern Denmark and the Danish International Development Agency (DANIDA).

## References

[CIT0001] Ai A.L, Peterson C, Ubelhor D (2002). War-related trauma and symptoms of posttraumatic stress disorder among adult Kosovar refugees. Journal of Traumatic Stress.

[CIT0002] Akaike H (1987). Factor analysis and AIC. Psychometrika.

[CIT0003] American Psychiatric Association (2013). Diagnostic and Statistical Manual of Mental Disorders.

[CIT0004] Amone-P'Olak K, Jones P.B, Abbott R, Meiser-Stedman R, Ovuga E, Croudace T.J (2013). Cohort profile: Mental health following extreme trauma in a northern Ugandan cohort of War-Affected Youth Study (The WAYS Study). SpringerPlus.

[CIT0005] Amone-P'Olak K, Jones P.B, Meiser-Stedman R, Abbott R, Ayella-Ataro P.S, Amone J., …, Ovuga E (2014). War experiences, general functioning and barriers to care among former child soldiers in Northern Uganda: The WAYS study. Journal of Public Health.

[CIT0006] Bayer C.P, Klasen F, Adam H (2007). Association of trauma and PTSD symptoms with openness to reconciliation and feelings of revenge among former Ugandan and Congolese child soldiers. JAMA.

[CIT0007] Betancourt T.S, Bass J, Borisova I, Neugebauer R, Speelman L, Onyango G., …, Bolton P (2009). Assessing local instrument reliability and validity: A field-based example from northern Uganda. Social Psychiatry and Psychiatric Epidemiology.

[CIT0008] Betancourt T.S, Borisova I, Williams T.P, Meyers-Ohki S.E, Rubin-Smith J.E, Annan J., …, Kohrt B.A (2013). Research review: Psychosocial adjustment and mental health in former child soldiers—A systematic review of the literature and recommendations for future research. Journal of Child Psychology and Psychiatry.

[CIT0009] Catani C, Jacob N, Schauer E, Kohila M, Neuner F (2008). Family violence, war and natural disasters: A study of the effect of extreme stress on children's mental health in Sri Lanka. BMC Psychiatry.

[CIT0010] Christiansen D.M, Elklit A, Lazinica A, Ovuga E (2012). Sex differences in PTSD. Posttraumatic stress disorder in a global context.

[CIT0011] Cloitre M, Garvert D.W, Brewin C.R, Bryant R.A, Maercker A (2013). Evidence for proposed ICD-11 PTSD and complex PTSD: A latent profile analysis. European Journal of Psychotraumatology.

[CIT0012] Cloitre M, Roberts N.P, Bisson J.I, Brewin C.R (2015). The ICD-11 Trauma Questionnaire.

[CIT0013] Child Soldiers. Global Report (2008). Coalition to stop the use of child soldiers.

[CIT0014] Cody M.W, Jones J.M, Woodward M.J, Simmons C.A, Beck J.G (2015). Correspondence between self-report measures and clinician assessments of psychopathology in female intimate partner violence survivors. Journal of Interpersonal Violence.

[CIT0015] Elklit A, Hyland P, Shevlin M (2014). Evidence of symptom profiles consistent with posttraumatic stress disorder and complex posttraumatic stress disorder in different trauma samples. European Journal of Psychotraumatology.

[CIT0016] Ertl V, Pfeiffer A, Schauer E, Elbert T, Neuner F (2011). Community-implemented trauma therapy for former child soldiers in northern Uganda: A randomized controlled trial. JAMA.

[CIT0017] Gavranidou M, Rosner R (2003). The weaker sex? Gender and posttraumatic stress disorder. Depression and Anxiety.

[CIT0018] Herman J.L (1992). Complex PTSD. A syndrome in survivors of prolonged and repeated trauma. Journal of Traumatic Stress.

[CIT0019] Hinton D, Hsia C, Um K, Otto M.W (2003). Anger-associated panic attacks in Cambodian refugees with PTSD; a multiple baseline examination of clinical data. Behaviour Research and Therapy.

[CIT0020] Johnson H, Thompson A (2008). The development and maintenance of posttraumatic stress disorder (PTSD) in civilian adult survivors of war trauma and torture: A review. Clinical Psychology Review.

[CIT0022] Klasen F, Reissmann S, Voss C, Okello J (2015). The guiltless guilty: Trauma-related guilt and psychopathology in former Ugandan child soldiers. Child Psychiatry & Human Development.

[CIT0023] Knefel M, Garvert D.W, Cloitre M, Lueger-Schuster B (2015). Update to an evaluation of ICD-11 PTSD and complex PTSD criteria in a sample of adult survivors of childhood institutional abuse by Knefel & Lueger-Schuster (2013): A latent profile analysis. European Journal of Psychotraumatology.

[CIT0024] Lo Y, Mendell N.R, Rubin D.B (2001). Testing the number of components in a normal mixture. Biometrika.

[CIT0025] Maercker A, Brewin C.R, Bryant R.A, Cloitre M, Reed G.M, Van Ommeren M, Saxena S (2013a). Proposals for mental disorders specifically associated with stress in the International Classification of Diseases-11. The Lancet.

[CIT0026] Maercker A, Brewin C.R, Bryant R.A, Cloitre M, Van Ommeren M, Jones L.M, Reed G.M (2013b). Diagnosis and classification of disorders specifically associated with stress: Proposals for ICD-11. World Psychiatry.

[CIT0027] McCutcheon A.C (1987). Latent class analysis.

[CIT0028] McLachlan G.J, Peel D (2000). Finite mixture models.

[CIT0029] Miller K.E, Rasmussen A (2010). War exposure, daily stressors, and mental health in conflict and post-conflict settings: Bridging the divide between trauma-focused and psychosocial frameworks. Social Science & Medicine.

[CIT0030] Mugisha J, Muyinda H, Wandiembe P, Kinyanda E (2015). Prevalence and factors associated with posttraumatic stress disorder seven years after the conflict in three districts in northern Uganda (The Wayo-Nero Study). BMC Psychiatry.

[CIT0031] Muthen L.K, Muthen B.O (1998–2012). Mplus user's guide.

[CIT0032] Nylund K.L, Asparouhov T, Muthén B.O (2007). Deciding on the number of classes in latent class analysis and growth mixture modeling: A Monte Carlo simulation study. Structural Equation Modeling.

[CIT0033] Orth U, Wieland E (2006). Anger, hostility, and posttraumatic stress disorder in trauma-exposed adults: A meta-analysis. Journal of Consulting and Clinical Psychology.

[CIT0034] Owens G.P, Chard K.M, Cox T (2008). The relationship between maladaptive cognitions, anger expression, and posttraumatic stress disorder among veterans in residential treatment. Journal of Aggression, Maltreatment & Trauma.

[CIT0035] Rees S, Silove D, Verdial T, Tam N, Savio E, Fonseca Z, Brooks R (2013). Intermittent explosive disorder amongst women in conflict affected Timor-Leste: Associations with human rights trauma, ongoing violence, poverty, and injustice. PLoS One.

[CIT0036] Resick P.A, Bovin M.J, Calloway A.L, Dick A.M, King M.W, Mitchell K.S., …, Wolf E.J (2012). A critical evaluation of the complex PTSD literature: Implications for DSM-5. Journal of Traumatic Stress.

[CIT0037] Roberts B, Ocaka K.F, Browne J, Oyok T, Sondorp E (2008). Factors associated with posttraumatic stress disorder and depression amongst internally displaced persons in Northern Uganda. BMC Psychiatry.

[CIT0038] Rosner R, Powell S, Butollo W (2003). Posttraumatic stress disorder three years after the siege of Sarajevo. Journal of Clinical Psychology.

[CIT0039] Schwarz G (1978). Estimating the dimension of a model. The Annals of Statistics.

[CIT0040] Sclove S.L (1987). Application of model-selection criteria to some problems in multivariate analysis. Psychometrika.

[CIT0041] Tay A.K, Rees S, Chen J, Kareth M, Silove D (2015). The structure of posttraumatic stress disorder and complex posttraumatic stress disorder amongst West Papuan refugees. BMC Psychiatry.

[CIT0042] UNICEF (2010). B&H Postwar Screening Survey.

[CIT0043] Wolf E.J, Miller M.W, Kilpatrick D, Resnick H.S, Badour C.L, Marx B.P, Friedman M.J (2015). ICD–11 Complex PTSD in US National and Veteran Samples New York: UNICEF. Prevalence and Structural Associations with PTSD. Clinical Psychological Science.

[CIT0044] Yuan K.H, Bentler P.M (2000). Three likelihood-based methods for mean and covariance structure analysis with non-normal missing data. Sociological Methodology.

